# Human herpes virus type-6 is associated with central nervous system infections in children in Sudan

**DOI:** 10.4102/ajlm.v11i1.1718

**Published:** 2022-09-22

**Authors:** Nada A. Abdelrahim, Nahla Mohamed, Magnus Evander, Clas Ahlm, Imad M. Fadl-Elmula

**Affiliations:** 1 Department of Medical Microbiology, Faculty of Medical Laboratory Sciences, Nile University, Khartoum, Sudan; 2 Department of Virology, Faculty of Clinical Microbiology, Umeå University, Umeå, Sweden; 3 Department of Infection and Immunology, Faculty of Clinical Microbiology, Umeå University, Umeå, Sweden; 4 Department of Pathology and Clinical Genetics, Faculty of Medicine, Al-Neelain University, Khartoum, Sudan; 5 Assafa Academy, Kartoum, Sudan

**Keywords:** HHV-6, viral neuroinfections, viral meningitis, aseptic meningitis, real-time PCR

## Abstract

**Background:**

Human herpes virus type-6 (HHV-6) is increasingly recognised as a febrile agent in children. However, less is known in sub-Saharan African countries, including Sudan.

**Objective:**

We investigated the involvement of HHV-6 in paediatric central nervous system (CNS) infections in Khartoum, Sudan.

**Methods:**

Febrile patients aged up to 15 years with suspected CNS infections at Omdurman Hospital for Children from 01 December 2009 to 01 August 2010 were included. Viral DNA was extracted from leftover cerebrospinal fluid (CSF) specimens and quantitatively amplified by real-time polymerase chain reaction (PCR) at Umeå University in Sweden.

**Results:**

Of 503 CSF specimens, 13 (2.6%) were positive for HHV-6 (33.0% [13/40 of cases with proven infectious meningitis]). The median thermal cycle threshold for all HHV-6-positive specimens was 38 (range: 31.9–40.8). The median number of virus copies was 281.3/PCR run (1 × 10^5^ copies/mL CSF; range: 30–44 × 10^3^ copies/PCR run [12 × 10^3^ – 18 × 10^6^ copies/mL CSF]). All positive patients presented with fever and vomiting; 86.0% had seizures. The male-to-female ratio was 1:1; 50.0% were toddlers, 42.0% infants and 8.0% teenagers. Most (83.0%) were admitted in the dry season and 17.0% in the rainy season. Cerebrospinal fluid leukocytosis was seen in 33.0%, CSF glucose levels were normal in 86.0% and low in 14.0%, and CSF protein levels were low in 14.0% and high in 43.0%.

**Conclusion:**

Among children in Sudan with CNS infections, HHV-6 is common. Studies on the existence and spread of HHV-6 chromosomal integration in this population are needed.

## Introduction

Human Herpes Virus type-6 (HHV-6) is a major cause of acute febrile illnesses in young children^1^ where most are infected by the age of three.^2^ The virus remains latent in white blood cells (i.e. monocytes and macrophages) following a primary infection and persistence in salivary glands.^3^ Reactivation of HHV-6 can occur in the case of an immunosuppression which may result in complications affecting various systems including the central nervous system (CNS).^3^ Young immunocompetent children who suffer from fever and seizures can develop CNS disease in primary HHV-6 infections.^4,5^ In this matter, several authors have described the major role that could be played by the virus as a cause of paediatric neuroinfections.^6,7,8,9,10,11^

Detecting HHV-6 nucleic acids in the cerebrospinal fluid (CSF) by molecular assays indicates active virus replication, hence CNS infection. This interpretation is complicated by the phenomenon of HHV-6 chromosomal integration.^12,13^ The only human herpes virus that is found integrated in the human genome and can be passed on vertically from parent to child is HHV-6.^14^ This occurs occasionally, and is claimed to be detected by simply identifying persistently high concentrations of HHV-6 nucleic acids in blood because of chromosomal integration in white blood cells.^12,13^ In contrast, integrated HHV-6 DNA is highly unexpected in normal cell-free body compartments, including the CSF.^11,13,15^ Ward^16^ stated that viral load should be high to identify a condition as chromosomal integration and low virus copies would indicate an infection.

Normal CSF glucose with normal or elevated proteins is the usual finding in viral infections of the CNS.^17^ Increased numbers of white blood cells (˃ 5 cell/mm^3^) in CSF indicates inflammation; however, normal CSF cellular counts in patients with proven CNS infections have been frequently reported.^17,18,19,20^ It is therefore recommended not to limit HHV-6 testing to patients with increased CSF cellular counts.^21^ Most patients recover fully from an HHV-6 infection, but rare complications leading to neurological impairments or death can also occur.^22,23^ Little is known on HHV-6 infections in Saharan and sub-Saharan Africa, and the infection has never been investigated in Sudan. We, therefore, intended to identify the involvement of HHV-6 in CNS infections in a large group of children in Khartoum, Sudan. This report is a part of a larger study on the microbial aetiologies of CNS infections in this population.

## Methods

### Ethical considerations

The ethical clearance for conducting this study was obtained from the Ethical Review Board of the National Center for Neurological Sciences in September 2009. Patient consent was determined to be unnecessary and was waived. Patients were not contacted directly; data were obtained from hospital files and were kept anonymous at all stages of the study. Excess specimens were obtained from the hospitals main laboratory after all officially requested tests were applied. Permission to collect data and specimens was granted from hospital authorities verbally (from hospital general director; authors’ attestation on file) and in writing (from Laboratories Administration, Federal Ministry of Health).

### Study materials

A total of 503 CSF specimens were obtained from febrile (˃ 37 °C) meningitis-suspected attendees of the Omdurman Hospital for Children in Khartoum, Sudan. Patients aged 0 to 15 years who were admitted between 01 December 2009 and 01 August 2010 were included. Clinical and demographic data were obtained from hospital records retrospectively. Routine CSF analyses were performed at the Microbiology Laboratory of Omdurman Hospital. An aliquot of CSF was frozen at −80 °C for further analysis for HHV-6 DNA at the Department of Clinical Microbiology, Umeå University, Umeå, Sweden. Human Herpes Virus type-6 DNA was extracted then quantitatively amplified by real-time polymerase chain reaction (PCR) using TaqMan^®^ (Eurogentec®, Seraing, Liège, Belgium) method.

### Viral DNA extraction

The QIAamp UltraSens Virus Technology Kit (Qiagen^®^, Hilden, Germany) was used for viral DNA extraction in CSF. One mL specimen was used to concentrate viral DNA: buffer AC (Qiagen^®^, Hilden, Germany) was added, shortly incubated, then sediment by low *g*-force (i.e. 1000–1200 × g) centrifugation (Eppindorf Centrifuge 5415D^®^, Hamburg, Germany) to pellet nucleic acid complexes. The supernatant was discarded; the pellet was re-suspended in buffer AR (Qiagen^®^, Hilden, Germany) and proteinase K and incubated for 10 min at 40°C. Binding conditions were adjusted by adding buffer AB (Qiagen^®^, Hilden, Germany); the lysate was applied to the silica gel membrane spin column. During brief centrifugation (i.e. 3000–5000 × g), DNA selectively binds to the membrane. Remaining contaminants and enzyme inhibitors were removed by centrifugation (i.e. 3000–5000 × g) in two wash steps using buffer AW1 then buffer AW2. Pure viral nucleic acids were eluted in 30 µL low-salt buffer AVE (Qiagen^®^, Hilden, Germany) twice. Each elute (60 µL) was divided into two aliquots (30 µL each) and preserved at −80 °C.

### TaqMan^®^ real-time polymerase chain reaction

**FIGURE 1:**
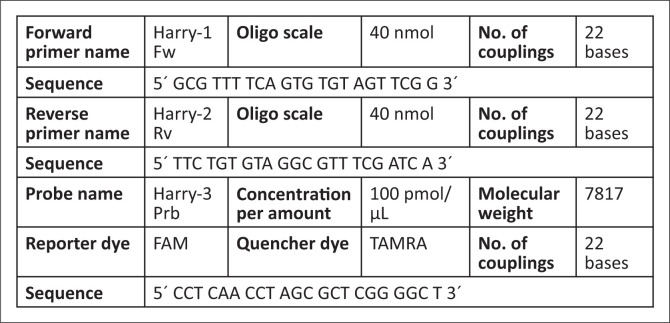
Specifications of human herpes virus type 6 primers and probe in Khartoum, Sudan, December 2009 – August 2010.

Viral gDNA amplification and detection was performed by real-time analysis using the Applied Biosystems^®^ 7900HT Fast Real-Time PCR system (Foster City, California, United States) and the TaqMan^®^ (Eurogentec^®^, Seraing, Liège, Belgium) probe (reporter dye FAM^TM^ on 5´ end and quencher dye TAMRA^TM^ on 3´ end). The lower assay detection limit is one virus copy per mL specimen. Forward and reverse primers (Figure 1) and probes (DNA Technology^®^, Aarhus, Denmark) were diluted to reach final working concentration of 25 µM (standardised concentration by Umeå University Hospital). Commercially provided oligonucleotide products were diluted to the suitable working solution and the recommendation of QuantiTect^®^ (Qiagen^®^, Hilden, Germany) Q PCR Protocol was followed. A PCR master mix solution was prepared for *Harry* (HHV-6) primers (Figure 1) as follow: volumes of 12.5 µL of 2 × QuantiTect^TM^ Probe PCR Master Mix (Qiagen^®^, Hilden, Germany), 1.0 µL forward primer (final concentration 25 µM), 1.0 µL reverse primer (final concentration 25 µM), 0.8 µL probe (final concentration 20 µM) and 7.2 µL rNase-free water (Ambion^®^, Waltham, Massachusetts, United States) were added into 2.5 µL template gDNA to complete a total reaction volume of 25.0 µL per single PCR reaction. The mixture was pulse-vortexed (Vortex-Genie Pulse^®^, Bohemia, New York, United States) and centrifuged briefly (i.e. 3000 × g). Then, 22.5 µL single reaction mix was transferred into a well of a MicroAmp^TM^ Optical 96-Well Reaction Plate (Applied Biosystems^®^, Foster City, California, United States) to which 2.5 µL template gDNA was added to reach total volume of 25.0 µL per one reaction.

The PCR plates were covered by MicroAmp^TM^ Optical Adhesive Film (Applied Biosystems^®^), concentrated at the bottom of the plate wells by spinning at low speed in a cold centrifuge (i.e. 1200 × g) using Allegra^TM^ X-12R Centrifuge (Beckman Coulter^®^, Brea, California, United States). Each PCR reaction plate was designed for carrying eight standard dilutions, one negative reverse transcriptase preparation, one non-template control containing ddH_2_O and duplicates or triplicates of each experimental DNA template.

The standard used for PCR had 5 × 10^6^ (5E6) virus copies (obtained from the Virology Department, Umeå University Hospital). The first standard preparation was used without dilution, having a concentration of 5E6 copies, followed by 1/10 serial dilutions for the subsequent seven preparations to reach final concentrations of 5E5, 5E4, 5E3, 5E2, 5E1, 5 and 1 virus copy.

Real-time cycler thermal condition was as follows: heating at 50 °C for 2 min, initial activation of HotStarTaq^®^ DNA Polymerase (Qiagen^®^, Hilden, Germany) at 95 °C for 10 min, denaturation at 94 °C for 15 s and combined annealing and extension at 60 °C for 60 s. The cycle was repeated 45 times taking an approximate duration of 90 min including generation of amplification curve. Data were analysed using the software ABI 7900HT SDS Plate Utility Version 2.4 (Applied Biosystems^®^).

### Statistical analysis

Statistical package program Statistical Package for Social Sciences version 21 (IBM Corp., Chicago, Illinois, United States) was used. Categorical variables were expressed in frequencies and percentages and cross-tabulated and Pearson chi-square tested for statistically significant differences under the 0.05 level. Numerical variables were described using measures of central tendency and of dispersion; Pearson’s correlation and its 95% confidence intervals were calculated.

## Results

### Clinical, demographic and conventional laboratory findings

**FIGURE 2:**
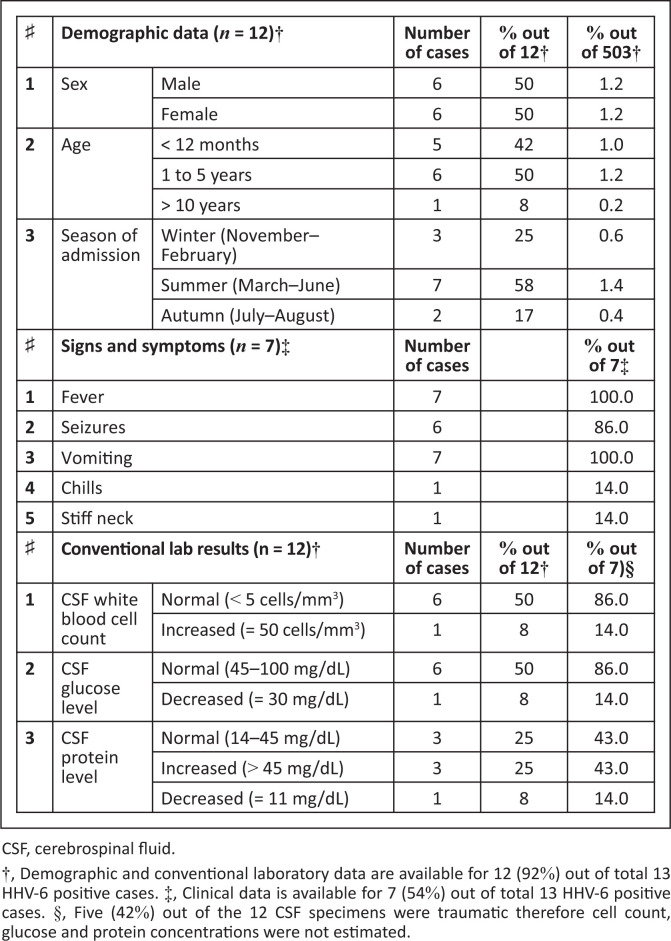
Demographic, clinical and conventional laboratory data for Human Herpes Virus type-6 positive patients, Khartoum, Sudan, December 2009 – August 2010.

All of the following describe patients with positive CSF HHV-6 DNA: all patients with clinical data (100%; 7/7; six cases out of a total of 13 had missing clinical data) presented with fever (˃ 37 °C) and vomiting and six (86%) with seizures (Figure 2). For the 12 patients with available demographic data, the male-to-female ratio was 1:1 (Figure 2).

Half of the patients were toddlers, followed by 42% (5/12; 1 missing case) infants and 8% (1/12) a 15-year-old teenager. Most patients (83%; 10/12) were admitted to the hospital in the dry season (December to June) and the remaining 17% (2/12) admitted in the rainy season (July to August). Cerebrospinal fluid specimens from 42% (5/12) were traumatic; cytological and chemical analyses were not performed. The remaining 58% (7/12) were clear; 33% (1/7) showed increased CSF white blood cells (50 cells/mm^3^) with 60% lymphocytosis and the remaining 67% (6/7) showed normal CSF white blood cell count (< 5 cells/mm^3^). Cerebrospinal fluid glucose levels for most specimens (86%; 6/7) were normal (45 mg/dL – 100 mg/dL) and one specimen (14%) was low (30 mg/dL). Cerebrospinal fluid proteins levels were high (> 45 mg/dL) in 43% (3/7), normal (14 mg/dL – 45 mg/dL ) in 43% (3/7) and low (11 mg/dL) in 14% (1/7) (Figure 2). All 13 HHV-6 positive cases did not show evidence of rapid-growing-bacteria coexisting in CSF on Gram’s stain and in vitro bacterial culture. However, 23% (3/13) were positive for other non-cultivable microbes. All patients (100%; 7/7) recovered and were discharged.

### Real-time polymerase chain reaction findings

Polymerase chain reaction testing for HHV-6 DNA in CSF revealed 13 (2.6%) positive specimens out of a total of 503. Median cycle threshold (Ct) for all HHV-6 positive specimens was 38 with a range of 31.9 to 40.8. Median virus copy was 281.3 per PCR run (1 × 10^5^ virus copies/mL CSF) with a range of 30 to 44 × 10^3^ copies per PCR run (12 × 10^3^ and 18 × 10^6^ virus copies/mL CSF). Individual PCR data for all 13 positive specimens are shown in Table 1. Standard dilutions with viral copies of 5E6 to 5E2 produced amplification curves between Ct 24 and Ct 36. The generated standard curve plot showed perfect negative association between Ct and viral quantity. This was repeatable in all PCR runs.

**TABLE 1:** Thermal cycle and virus load for Human Herpes Virus type-6 positive individual cerebrospinal fluid specimens using quantitative TaqMan real-time polymerase chain reaction, Khartoum, Sudan, December 2009 – August 2010.

Sample ID	Cycle threshold	Virus quantity per PCR run
1	38.03	32 × 10^3^
2	39.37	1 × 10^2^
3	40.83	30
4	37.31	527
5	40.13	54
6	38.38	221
7	38.09	281
8	34.99	3.5 × 10^3^
9	37.64	69
10	36.77	818
11	40.59	37
12	35.65	2 × 10^3^
13	31.87	44 × 10^3^

PCR, polymerase chain reaction.

The final classification of cases based on clinical and molecular findings is shown in Table 2.

**TABLE 2:** Classification of cases based on clinical and molecular findings, Khartoum, Sudan, December 2009 – August 2010.

Defined groups	Cases out of 503†		% out of 40‡
	%	Frequency	
Proven infectious meningitis§	8.0	40‡	100
Proven viral meningitis	3.2	16¶	40
HHV-6 meningitis	2.6	13	33

† , Total number of febrile suspected patients who attended the hospital during the study period and were subjected to lumbar puncture.

‡ , 17 cases with positive microbial origin – but with normal cellular count – along with all 23 cases with cerebrospinal fluid pleocytosis with or without positive microbial aetiology.

§ , This information has been shared in our previous publication.^20^

¶ , 13 cases with confirmed Human Herpes Virus type-6 DNA and 3 cases with other confirmed viral nucleic acids.

## Discussion

Human Herpes Virus type-6 is becoming increasingly recognised as an emerging CNS pathogen; nevertheless, it has never been investigated in Sudan and very little is known about it in sub-Saharan African countries or those of the meningitis belt. In the present study, HHV-6 DNA was identified in the CSF of 2.6% of paediatric patients with suspected meningitis. This result is close to the findings of Hosseininasab^24^ who identified CSF HHV-6 DNA in 1.5% (1/65) of children with aseptic meningitis in Southern Iran. Tavakoli^21^ reported similar findings of 1.8% (27/1482) in New York, United States. Another study in New York by Yao^11^ revealed a significantly higher prevalence of 40.0% (14/35) in patients with CNS infections who tested negative for other CNS pathogens. Among well-defined groups in our study, the prevalence was also high, accounting for 33.0% out of 40 cases with proven infectious meningitis (Table 2). In the present study, unlike Yao’s approach, cases with other detectable CNS pathogens were not excluded because of possible co-infections, as frequently reported.^21,25,26,27^

It has been reported that primary infection with HHV-6 always occurs in young children (age 0 to 2 years).^5,28,29^ Human Herpes Virus type-6 infections, especially to the CNS, can also occur in healthy older children and adults, but it is thought to be due to virus reactivation.^22^ Among 24 HHV-6 positive patients in Tavakoli’s study,^21^ 42% were infants ≤ 3 years and 12.5% were teenagers 11–17 years old. Out of our 13 HHV-6 positive cases, 92% were infants ≤ 2.3 years and one (8%) was a 15-year-old teenager. The ratio of boys to girls in this study was 1:1 which agrees with Tavakoli’s findings of 1.3:1.1.

Clinical signs and symptoms in HHV-6 meningitis are not specific.^30^ Out of the 24 examined patients, Tavakoli reported fever in 71.0%, altered mental status in 67.0%, headache in 29.0% and seizures in 33.0%. Other reported symptoms were muscle weakness, muscle pain and stiff neck, which are general symptoms for meningitis or encephalitis.^21^ In the present study, all HHV-6 positive cases presented with fever and vomiting, 86.0% presented with seizures, 14.3% with chills and 14.3% with a stiff neck. None of our patients developed skin rash, which is the only specific – but rare – symptom in HHV-6 meningitis.^30^ Findings of both Tavakoli and Hosseininasab concur.

Normal CSF glucose with normal or elevated proteins is the usual finding in viral infections of the CNS,^17^ as found in this study, where all specimens showed normal CSF glucose concentration, 33% normal proteins and 67% high proteins. Unfortunately, chemical analysis of the CSF is ineffective in case of viral infections; however, its significance is in distinguishing bacterial from aseptic aetiologies which is a crucial preliminary step in deciding adequate treatment. Cerebrospinal fluid leukocytosis was seen in 33%. Increased CSF white blood cell numbers indicate inflammation; however, normal CSF cellular counts do not rule out viral aetiologies. In fact, normal CSF cellular counts in patients with proven CNS infections were frequently reported.^17,18,19,20^ Normal CSF profile was reported in 25% of HHV-6 positive cases in Tavakoli’s study^21^; accordingly, HHV-6 testing should not be limited to patients with abnormal CSF profiles.

Thermal Ct is defined as the number of cycles required for the fluorescent signal to cross the threshold (i.e. exceed background level). Median Ct for our positive cases was 38 with a range of 31.9 to 40.8. In Tavakoli’s study, Ct values ranged from 25.03 to 39.92. In quantitative real-time PCR, Ct values inversely correlate with viral loads; therefore, a low Ct value indicates a high viral load and vice versa. Our Ct values and viral loads were found to be significantly (*p* = 0.029) inversely correlated (*r* = −0.6, 95% confidence interval: −0.1 to −0.9) indicating significant variation of viral loads among our patients. Substantial variation among viral loads in patients was also reported by Tavakoli.^21^

The phenomenon of HHV-6 chromosomal integration is in debate; while some^11,12,13^ consider it an easily identifiable condition based on the presence or absence of nucleus containing blood cells in different body compartments, Ward^16^ believes the few leukocytes that are usually present in normal CSF can reveal positive viral DNA in the case of HHV-6 integration. In the present study, the CSF was clear with no observed cells in most cases (86%), while a single case (14%) showed an increased CSF cell count. Ward^16^ elaborated that in order to identify a condition as chromosomal integration, viral load should be high while low virus copies would indicate an infection. They^16^ reported a significantly lower (i.e. 2.4 log10 copies/mL) CSF HHV-6 DNA concentration in 9 children with primary infection in comparison with 21 patients with viral chromosomal integration (i.e. 4.0 log10 copies/mL). In the present study, the median CSF virus concentration was 1 × 10^5^ copies per mL with a minimum virus concentration of 12 × 10^3^ copies per mL and a maximum of 18 × 10^6^ copies per mL. While Ward^16^ recommended identifying low virus copies (≤ 10^3^) as an acute HHV-6 infection and high virus copies (≥ 10^4^) as chromosomal integration, Collot^31^ identified viral integration in approximate concentrations of 10^3^ to 10^6^ copies per mL. In the present study, we identified viral concentrations as low as 10^4^ and as high as 10^7^. Several other studies reported high CSF viral loads in patients with HHV-6 CNS infections.^11,21^ In addition, other authors^12,13^ insist that chromosomal integration is a rare condition. Accordingly, we assume the detected HHV-6 DNA in our mostly cell-free CSF specimens is more likely to be from free replicating virus than from chromosomally integrated virus.

In infants, primary HHV-6 infection is an important cause of febrile seizures with an incidence of 13% in the United States.^4^ Knowing that febrile seizures and vomiting were dominant symptoms among our population and the most frequent age group was children up to the age of 2.3 years, therefore, further supported our assumption. Despite this, and for the sake of scientific relevance, we are not ruling out the possibility of integration among our identified cases. For this reason, studies to identify the prevalence of HHV-6 integration among healthy Sudanese population are warranted.

Alongside HHV-6, mixed microbial infections were identified in three cases in this study. Several authors also reported mixed viral infections to the CNS.^21,25,26,27^ Despite moderate neurological sequel, and less likely death, patients usually recover fully from an HHV-6 infection^22,23^, as fortunately observed in this population.

### Limitations

A major limitation in this study, however, is that we were unable to further genotype our identified HHV-6 viral DNA because of a limited CSF volume (i.e. all available CSF was consumed in testing and identifying multiple microbes – reported in other publications).

### Conclusion

Human Herpes Virus type-6 CNS infection is frequent in this population (i.e. identified in one-third of cases with proven infectious meningitis). We recommend studying the existence and spread of HHV-6 chromosomal integration in the healthy Sudanese population.
